# Randomized Trial of a Training Program to Improve Home Visitor Communication around Sensitive Topics

**DOI:** 10.1007/s10995-018-2531-0

**Published:** 2018-05-31

**Authors:** Allison West, Laina Gagliardi, Amanda Gatewood, Susan Higman, Jane Daniels, Kay O’Neill, Anne Duggan

**Affiliations:** Department of Population, Family and Reproductive Health, Johns Hopkins Bloomberg School of Public Health, Baltimore, USA

**Keywords:** Home visiting, Training evaluation, Communication, Fidelity, Motivational interviewing

## Abstract

**Introduction:**

Strong communication skills are necessary to engage families, perform accurate assessments, and motivate behavior change around sensitive issues encountered in home visiting.

**Methods:**

A two-arm, cluster-randomized trial evaluated the impact of a trans-model communications training course for home visitors. Fourteen home visiting programs in Maryland were assigned to a training intervention (*n* = 7 programs; 30 visitors) or wait-list control group (*n* = 7 programs; 34 visitors). Independent observers assessed training fidelity. Visitor’s attitudes, knowledge, and confidence were assessed through surveys. Their skills were assessed through coding of video-recorded visits with standardized mothers. Data were collected at baseline, within 2 weeks post-training, and at 2 months post-training. Regression models accounted for clustering within programs and controlled for characteristics on which study groups differed at baseline.

**Results:**

Independent observers rated the training highly on fidelity and acceptability. Home visitors rated it as useful, consistent with their model, and worth the effort. Immediately following the training, the training group scored higher than the control group on a range of indicators in all domains—knowledge, attitudes, confidence, and skills in using motivational communication techniques. At 2 months post-training, impacts on knowledge and attitudes persisted; impacts on confidence and observed skill were attenuated.

**Discussion:**

The training course showed favorable immediate impacts on knowledge, attitudes, confidence, and skills, and long-term impacts on home visitor knowledge and attitudes. The findings underscore the need for ongoing reinforcement of skills following training.

## Significance

To our knowledge, this study is the first to use standardized mothers to assess observed changes in home visitor skill following a communication skills training course. The findings provide empirical support for the acceptability and effectiveness of a trans-model approach to teaching motivational communication skills for use by home visitors when talking with families about sensitive issues.

## Introduction

Workforce development is a high priority in evidence-based home visiting. Building a stable, competent workforce is one of the top ten priorities on the national home visiting research agenda (Home Visiting Research Network 2013) and one of HRSA’s four focal areas for the federal Maternal, Infant, and Early Childhood Home Visiting (MIECHV) Innovation Awards (Health Resources and Services Administration 2016). Staff selection, training, and coaching are key elements of effective implementation systems to assure that staff are competent to carry out their roles effectively (Fixsen et al. 2005). Results from a recent meta-analytic review showed that the quality of home visitor training and supervision predicted program outcomes (Casillas et al. 2016).

One important area of workforce development concerns home visitors’ ability to communicate effectively with high risk families around sensitive issues. Strong communication skills are essential to engage and earn the trust of families, perform accurate assessments, and motivate behavior change (Frankel 2001). Most evidence-based home visiting programs seek to enroll families with multiple, complex needs in areas such as mental health, substance abuse, domestic violence, and poor parenting (Michalopoulous et al. 2015). Evidence suggests that home visitors find it difficult to discuss these topics with families (Jones Harden et al. 2010; Monteiro 2016; Tandon et al. 2005) and desire enhanced training and support to address sensitive issues effectively (Gill et al. 2007; Tandon et al. 2008).

Home visitor competence in addressing sensitive issues requires training and ongoing support to promote the use of skills in practice. Results from a survey conducted through the Home Visiting Applied Research Collaborative showed that home visiting programs expect home visitors to have strong communication skills and that they expect them to acquire these skills through training and other professional development activities after hire (Home Visiting Applied Research Collaborative 2017). All evidence-based home visiting models require pre-service and ongoing training (Sama-Miller et al. 2017). Most training is model-specific (Home Visiting Research Network 2013), although models and local implementing agencies often support or encourage home visitors to obtain additional training, certifications, or endorsements (e.g., Child Development Associate or Infant Mental Health endorsement). In addition, several states have developed core competencies for home visitors, and MIECHV Innovation Awards have been given to states developing training to support cross-model competencies (Health Resources and Services Administration 2017).

In 2016, the Maryland MIECHV program partnered with the University of Maryland, Baltimore County (UMBC) to develop and implement a communication skills certificate training course for home visitors, and with Johns Hopkins University to conduct an independent evaluation of the course. Course developers engaged home visiting models and local programs as partners in developing the training, which was aligned with principles of adult learning (Trivette et al. 2009), training transfer (Burke and Hutchins 2007; Grossman and Salas 2011), stages of change (DiClemente 2005), and motivational interviewing (Miller and Rollnick 2012). The course’s main objective was to build home visitor knowledge, attitudes, confidence, and skills to communicate with families around sensitive topics.

This manuscript reports findings from a two-arm cluster-randomized trial in which 14 home visiting programs were assigned to either the certificate training course or a wait-list control condition. The purpose of the study was to evaluate the fidelity, acceptability, and impacts of the training course on home visitors’ knowledge, attitudes, confidence, and communication skills around sensitive issues. We developed an innovative observational method, the standardized mother procedure, to assess home visitor skill.

## Methods

### Participants and Procedures

Maryland home visiting programs were eligible for study participation if they used an evidence-based model and served pregnant women and children under the age of three. Programs receiving MIECHV funding were given priority for study enrollment. Seven programs were excluded because they had participated in one of the two pilot versions of the training program. Two additional programs were excluded because they had recently participated in a similar training program. Home visitors at participating programs were eligible if they had active caseloads and could travel to the training location. Supervisors of eligible home visitors were also invited to participate to enhance their capacity to reinforce skills and support training transfer.

**Fig. 1:**
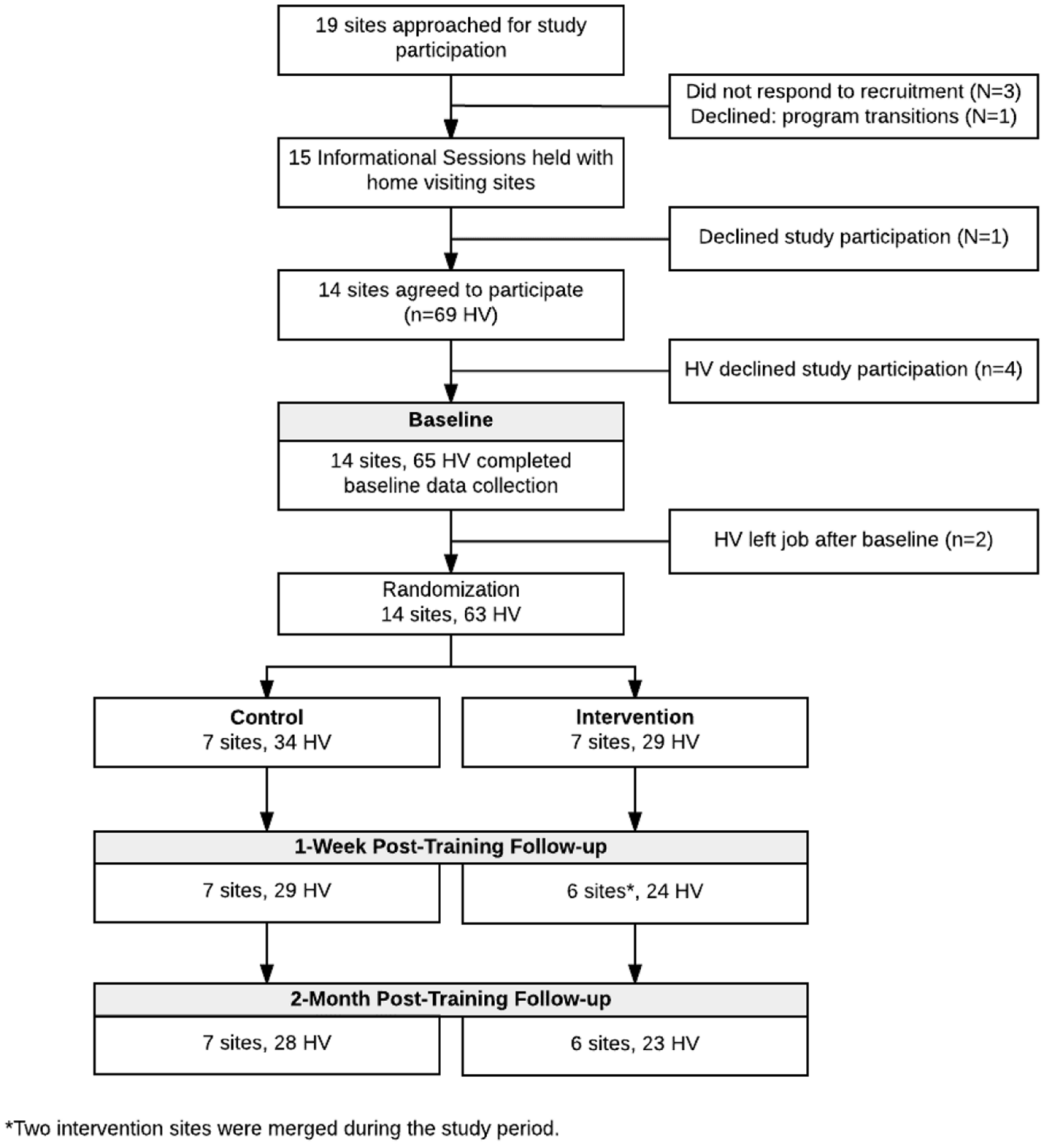
Consort diagram

A total of 19 programs were approached for study participation between June and August 2016 (Fig. 1). The study was first introduced at meetings of local home visiting programs, after which the Maryland Department of Health emailed programs additional study details. The study coordinator then scheduled web-based study information sessions with staff at each site. Three programs did not respond to recruitment contacts. One program declined participation due to program leadership transitions. Information sessions were held with 15 programs. One program declined participation after the informational session. Healthy Families America (HFA; *n* = 12) and Early Head Start home-based (EHS; *n* = 2) programs were randomly assigned to the training intervention (*n* = 7 programs; 29 visitors) or the wait-list control group (*n* = 7 programs; 34 visitors). Programs were randomized by site to acknowledge the shared characteristics and experiences among home visitors within programs and to reduce the possibility of contamination effects.

**Fig. 2:**
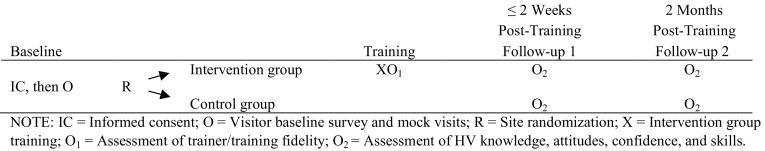
Study design

Data were collected at three points: pre-training baseline, within 2 weeks of training completion (follow-up 1), and at 2-months post-training (follow-up 2; Fig. 2). Research teams traveled to each program to obtain informed consent and collect data at each time point. Participants were remunerated for completing study activities. Study procedures were approved by the Institutional Review Boards of the Johns Hopkins Bloomberg School of Public Health and the Maryland State Department of Health.

### Training Intervention

Training developers used input from home visitors and supervisors to design the course. They solicited input through focus groups and individual interviews. In addition, trainers solicited feedback regularly from a stakeholder advisory board comprised of state partners, home visiting program staff, and content matter experts. Prior to the randomized trial, the course was tested and refined based on results from pilot pre- and post-tests and participant feedback on usefulness and acceptability.

The final course consisted of 6 modules delivered in 7 days over 12 weeks (Table 1). The objective of each module was to support participants’ development of core motivational communication competencies in each topic area. The first two training days were held back-to-back and focused on basic communication and listening skills, stages of change, and motivational interviewing techniques. The last five training days were held on a biweekly schedule. Participants who completed all modules received a certificate from UMBC’s Professional Studies program.

**Table 1:** Communication training course overview

Module	Length	Description of topic area
1. Communication	2 days	• Communication skills for difficult conversations with families • Stages of change and motivational interviewing
2. Healthy relationships	1 day	• Identifying signs of family distress, including domestic violence • Sensitive conversations around family conflict, safety planning
3. Parenting and child development	1 day	• Child development • Promoting positive parenting
4. Mental health	1 day	• Assessing mental health, supporting access to services, promoting coping • Techniques for staff to promote their own mental health
5. Substance use	1 day	• Addressing substance misuse • Screening and communication to promote behavior change and inform families about treatment and referral options
6. Cultural sensitivity	1 day	• Working with diverse populations

### Measures

#### Fidelity and Acceptability of Training

A study team member observed each training session to assess two dimensions of training fidelity: quality of delivery and participant responsiveness (James Bell Associates 2009). The observer rated trainers on ten aspects of quality on a Likert scale (1 = *low quality* to 5 = *high quality*), such as pace, rapport with learners, and responsiveness to trainees’ questions and concerns (adapted from Healthy Teen Network & RTI International 2017). The observer rated each trainee on two dimensions of participant responsiveness: understanding and participation (1 = *little understanding*/*low participation* to 5 = *good understanding*/*high participation*). To assess acceptability, trainees completed a four-item Likert scale at follow-up 2 to rate their perception that the program taught them skills that were helpful in their work, they could use easily in their work, were consistent with what their model teaches, and that the training was worth the effort (1 = *strongly disagree* to 4 = *strongly agree*). Items were summed to create on overall training acceptability score.

#### Home Visitor Characteristics, Attitudes, Knowledge, and Confidence

Home visitor characteristics, attitudes, knowledge, and confidence were assessed using self-administered surveys. Home visitor age, educational attainment, race, ethnicity, years of experience as a home visitor, and caseload size were assessed at baseline. Attitudes toward discussing parenting risks and concerns about parenting behaviors were assessed at all three time points. Three Likert-type items assessed home visitors’ attitudes toward discussing concerns about risks for poor parenting with parents (1 = *strongly disagree* to 4 = *strongly agree; α* = 0.68–0.76 across three time points). A sample item was, “Sharing concerns about a possible parenting risk is a sign of care and respect for the family.” Three additional items assessed attitudes toward discussing concerns about parenting behaviors (*α* = 0.47–0.54 across the three time points). A sample item was, “Keeping silent when observing harsh parenting behavior sends a signal that the behavior is acceptable.” Items were summed to create scale scores, with higher scores reflecting more positive attitudes.

Knowledge and confidence were assessed at the two follow-up points. Items were developed to align with course objectives and content and were reviewed by the trainers for content validity. The 36 knowledge items were presented on a 6-point Likert scale (1 = *strongly disagree* to 6 = *strongly agree*) and converted to dichotomous variables by recoding the disagree (1–3) and agree (4–6) response values as either correct or incorrect (scored as 1 or 0, respectively). Dichotomous values were added to generate a score (0–36) at each time point. To reduce testing effects, 17 new *True*/*False* knowledge items were added at follow-up 2; items were scored as correct (1) or incorrect (0). Thirteen confidence items were measured on a 7-point Likert scale (0 = *very strongly disagree* to 6 = *very strongly agree*); response values were added to generate a score (0–78) at each time point (*α* = 0.95 at follow-up 1 and 0.96 at follow-up 2). For ease of interpretation, knowledge and confidence scores were converted to scales ranging from 0 to 100, with higher scores reflecting greater knowledge and confidence, respectively.

#### Home Visitor Skill

Communication skill was assessed at all three time points using video-recorded “mock visits” with trained actresses serving as standardized mothers. The research team created six scenarios depicting the following sensitive issues: maternal depression, maternal smoking, maternal alcohol use, domestic violence, parenting/spanking, and maternal anxiety. Each scenario was designed to generate a 20–30 min conversation. Two mock visits were recorded with every visitor at each time point. Scenarios were paired to achieve variation in content, stage of change, and maternal reflective capacity (e.g., capacity to explore and link thoughts, feelings, and behavior).

Home visitors’ use of communication skills in mock visits was assessed in two ways. First, the standardized mothers provided global ratings of the home visitors’ skills immediately after each mock visit at follow-ups 1 and 2 by indicating their level of agreement with eleven statements on a 7-point Likert scale (1 = *strongly disagree* to 7 = *strongly agree*). Items were developed by the researchers or were adapted from instruments that assessed perceptions of the working alliance (Horvath and Greenberg 1994). Sample items included, “I sensed that the visitor was impatient or frustrated with me,” and “The visitor encouraged me to express my thoughts and feelings.” Items were summed to create a total score (*α* = 0.89). Standardized mothers were blind to group assignments and their ratings were not shared with the home visitors.

Second, trained research assistants coded the video-recorded mock visits using the Motivational Interviewing Treatment Integrity (MITI) Scale version 4.1 (Moyers et al. 2015). They coded the frequencies of ten specific behaviors (Give Information, Persuade, Persuade with Permission, Questions, Simple Reflection, Complex Reflection, Affirm, Seek Collaboration, Emphasize Autonomy, and Confront). The Questions code was adapted by making a distinction between Open- and Closed-ended Questions using conventions established in MITI 3.1 (Moyers et al. 2010). Four global scores (Cultivating Change Talk, Softening Sustain Talk, Partnership, & Empathy) were assigned using a 5-point Likert scale (1 = *low* to 5 = *high*). A technical global composite score was calculated by averaging cultivating change talk and softening sustain talk scores. A relational global composite score was calculated by averaging Partnership and Empathy scores. Composite scores were calculated for percentage of complex reflections, total instances of MI adherent behaviors (Affirm +Seek Collaboration + Emphasize Autonomy), and total instances of MI non-adherent behaviors (Persuade + Confront). Finally, three new scores were assigned to assess specific strategies that were emphasized in the training. These included two new global scores, Elicit-Provide-Elicit and Avoiding Labels and Stereotypes, and one new composite score that assessed the use of Open-ended questions, Affirmations, Reflections, and Summary Statements (OARS). MITI developers and trainers reviewed and approved adapted and new items for face and construct validity.

Coders included two undergraduate and three graduate students. Each received 27 h of formal training and participated in weekly and bi-weekly reliability meetings and assessments. Ten percent of videos were blind-coded by all coders to establish reliability. Intraclass Correlation Coefficients (ICCs) for 16 of 18 individual items were above 0.60, suggesting good or excellent agreement across coders (Cicchetti 1994). ICCs for avoiding labels and stereotypes and softening sustain talk were lower (0.50 and 0.13, respectively). The low ICC for softening sustain talk may be explained, in part, by low levels of sustain talk. Coders were blind to home visitors’ group assignments.

### Analytic Approach

Central tendency and variability in baseline characteristics, training fidelity and acceptability, and outcomes were examined using descriptive statistics. Minimal missing survey data (< 5%) were handled using ipsative mean imputation. There were no missing observational data. Characteristics of intervention and control group home visitors were compared for baseline equivalence using Chi square and t-tests. Variables with group differences at baseline were included as covariates in all models. To assess the impact of the training on outcomes, we used a conservative approach to multiple regression that adjusted for clustering of home visitors within the 14 home visiting programs. Models estimating attitudes and observed skills included baseline scores as covariates (knowledge and confidence were not assessed at baseline).

## Results

### Baseline Characteristics

The sample was diverse in demographic and work-related characteristics (Table 2). Home visitors randomized to the control group were similar to treatment group participants in race and ethnicity, educational attainment, years as a home visitor, attitudes toward talking with parents about parenting risks and behaviors, and observed communication skill. Participants randomized to the control group were slightly older and had smaller caseloads than home visitors in the treatment group.

**Table 2:** Baseline characteristics of home visitors by treatment group (N = 63)

Baseline characteristics	Control (*n* = 34)	Treatment (*n* = 29)	*p*
Age (mean, range)	40.2 (24–62)	34.9 (25–53)	.04
Race and ethnicity			.96
Black/African American	40%	41%	
White, non-Hispanic	30%	26%	
Hispanic/Latina	27%	30%	
Educational attainment			.83
High school/GED	1 (3%)	0 (0%)	
Some college/associates degree	11 (33%)	9 (31%)	
Bachelor’s degree	17 (52%)	18 (62%)	
Master’s degree	4 (12%)	2 (7%)	
Number of years as home visitor	6.3 (0.5–30)	3.3 (0.5–17)	.06
Number of families in caseload^*^	12.4 (4–19)	15.2 (0–24)	.05

### Fidelity and Acceptability

All but one trainee attended every module; one trainee missed the mental health module. Across all modules, independent observer ratings were very high for trainer fidelity (*Mean* = 4.8, *SD* = 0.2), trainees’ levels of understanding (*Mean* = 4.4; *SD* = 0.5) and trainees’ levels of participation (*Mean* = 4.0; *SD* = 0.7). At follow-up 2, trainees gave favorable ratings for acceptability (*Mean* = 3.6, *SD* = 0.5).

### Associations Between Knowledge, Attitudes, Confidence and Skill

Few consistent patterns of relationships among knowledge, attitudes, confidence, and observed skill were observed between follow-up 1 and follow-up 2. Knowledge was positively associated with more favorable attitudes toward talking about parenting risks and parenting behaviors at both time points. Knowledge was also associated with one observed behavior at both time points: Elicit-Provide-Plicit. Neither confidence nor attitudes showed any consistent pattern of association with observed skills across time.

### Program Impacts

#### Attitudes, Knowledge and Confidence

At follow-up 1, treatment group home visitors demonstrated more favorable attitudes than control group home visitors toward talking with parents about parenting risks but not toward talking with parents about their parenting behaviors (Table 3). At follow-up 1, the treatment group also exhibited higher levels of knowledge and confidence compared to the control group. Effects sizes ranged from medium to large (Cohen 1988). At follow-up 2, impacts on knowledge and attitudes endured, but the effects on confidence were attenuated and were no longer statistically significant.

**Table 3:** Immediate and long-term training effects on attitudes, knowledge and confidence

Outcome	Control adjusted mean (SD)	Treatment adjusted mean (SD)	Coefficient	Cohen’s *d*	*p*
Baseline (*N* = 53)					
Attitudes: talking about parenting risks	8.3 (2.7)	7.7 (3.1)	− 1.04	0.21	.07
Attitudes: talking about parenting behaviors	8.8 (2.4)	8.8 (2.0)	− 0.26	0	.63
Follow-up 1 (*N* = 53)					
Attitudes: talking about parenting risks	8.0 (2.9)	10.4 (1.5)	2.40	1.04	.01
Attitudes: talking about parenting behaviors	9.4 (1.9)	9.6 (1.9)	0.58	0.03	.22
Knowledge	76.1 (7.1)	85.1 (7.1)	7.94	1.26	.003
Confidence	74.5 (15.7)	83.5 (14.1)	8.98	0.60	.04
Follow-up 2 (*N* = 51)					
Attitudes: talking about parenting risks	8.6 (2.6)	9.5 (2.3)	1.28	0.38	.05
Attitudes: talking about parenting behaviors	9.3 (1.9)	9.4 (2.0)	0.20	0.06	.60
Knowledge	76.1 (9.1)	84.5 (8.5)	8.32	0.96	<.001
Knowledge supplement	74.6 (14.4)	82.8 (11.1)	8.77	0.64	.02
Confidence	76.0 (17.1)	80.3 (15.4)	4.52	0.26	.37

Scale scores for knowledge and confidence had possible range of 0–100. All models controlled for age and caseload size and were adjusted for clustering at the site level. Coefficients represent treatment group; control group is reference group. Models estimating training effects on attitudes controlled for baseline scores on attitude scales

#### Observed Skills

At follow-up 1, standardized mothers gave more favorable ratings of their interactions with training group home visitors compared to control group visitors (*p* = 0.04). At follow-up 2, this difference was no longer statistically significant. At follow-up 1, the training group demonstrated favorable scores relative to the control group on the adapted MITI (Moyers et al. 2015) for five of the eight observed communication skills. Effect sizes ranged from moderate to large (Table 4). The training showed largest impacts on relational global scores, use of the elicit-provide-elicit strategy, and avoidance of MI non-adherent strategies. By follow-up 2, most impacts were attenuated, although the effect of training on avoidance of MI non-adherent strategies endured.

**Table 4:** Immediate and long-term effects on observed skills

Outcome	Control adjusted mean (SD)	Treatment adjusted mean (SD)	Coefficient	Cohen’s *d*	*p*
Baseline (*N* = 53)					
Total MI adherent	4.7 (2.7)	3.9 (2.4)	− 0.64	− 0.32	.21
Total MI non-adherent	7.6 (4.9)	6.7 (4.3)	− 0.65	− 0.20	.62
Technical global	3.2 (0.5)	3.3 (0.5)	0.06	0.11	.65
Relational global	3.1 (0.6)	3.0 (0.7)	− 0.20	− 0.22	.34
Percent complex reflections	0.4 (0.2)	0.5 (0.2)	− 0.01	0.13	.80
Training-specific MITI adaptations					
Using elicit-provide-elicit strategy	2.7 (0.5)	2.5 (0.7)	− 0.24	0.28	.09
Open-ended questions, affirmations, reflections, summaries (OARS)	17.2 (8.3)	17.8 (5.3)	− 0.65	0.08	.70
Avoiding labels and stereotypes	4.0 (0.6)	4.1 (0.8)	0.05	0.14	.78
Follow**-**up 1 (*N* = 53)					
Total MI adherent	2.8 (2.3)	3.5 (2.3)	0.43	0.30	.38
Total MI non-adherent	7.5 (6.8)	3.2 (3.2)	− 3.59	− 0.80	.02
Technical global	3.4 (0.5)	3.6 (0.3)	0.21	0.56	.02
Relational global	3.3 (0.4)	3.9 (0.5)	0.51	1.24	<.001
Percent complex reflections	0.4 (0.2)	0.6 (0.2)	0.11	0.50	<.001
Training-specific MITI adaptations					
Using elicit-provide-elicit strategy	2.7 (0.6)	3.3 (0.7)	0.52	0.96	.002
OARS	18.8 (7.1)	18.7 (7.2)	0.74	− 0.01	.75
Avoiding labels and stereotypes	4.1 (0.5)	4.4 (0.5)	0.34	0.56	.08
Follow-up 2 (*N* = 51)					
Total MI adherent	3.0 (2.0)	3.1 (1.5)	0.09	0.05	.83
Total MI non-adherent	5.1 (4.4)	2.9 (2.3)	− 1.80	− 0.62	.04
Technical global	3.5 (0.4)	3.7 (0.3)	0.20	0.59	.20
Relational global	3.5 (0.6)	3.8 (0.5)	0.21	0.51	.19
Percent complex reflections	0.4 (0.2)	0.5 (0.2)	0.05	0.32	.47
Training-specific MITI adaptations					
Using elicit-provide-elicit strategy	2.9 (0.7)	3.3 (0.6)	0.24	0.56	.29
OARS	14.8 (6.2)	16.1 (6.2)	0.56	0.21	.82
Avoiding labels and stereotypes	4.3 (0.5)	4.5 (0.3)	0.14	0.32	.40

Coefficients represent treatment group; control group is reference group. All models controlled for baseline score, age, and caseload size and were adjusted for clustering at the site level

## Discussion

This cluster randomized trial found that a six-module course had favorable and consistent immediate impacts on home visitors’ communication knowledge, attitudes, confidence, and observed skills. The training produced the most enduring observed effect on reducing home visitors’ use of strategies that are incompatible with theories of motivational interviewing such as persuading or confronting parents who may not yet be open to behavior change. The findings suggest that home visitors with varying levels of education and experience could learn and apply motivational communication skills in simulated visits.

To our knowledge, this study is the first to use mock visits and ratings by standardized mothers to assess observed changes in skill following a communication skills training course for home visitors. Although more time intensive and costly, video-recorded observations are more objective than self-report measures of skill. In addition, mock visits offer two key advantages over recorded observations of real visits, particularly in research. First, mock visits are less intrusive and reduce concerns regarding family privacy and confidentiality. Second, mock visits allow for standardized presentation of the stimulus; thus, each home visitor encounters the same mother with the same presenting issues. This study demonstrated that mock visits are a feasible and effective way to assess variability in home visitor communication skills in response to scenarios depicting sensitive issues, and to assess change in skills over time.

The training’s impacts on confidence and observed skills diminished over time and did not remain consistent across outcomes. The findings underscore the importance of ongoing supervision, coaching, and other forms of ongoing reinforcement to facilitate the transfer of skills to practice (Burke and Hutchins 2007; DeRoten et al. 2013; Schwalbe et al. 2014). Closer examination of the data showed that long-term effects may have varied depending on the nature of the scenario. Thus, attenuated effects on skills may reflect variability in home visitors’ ability to apply skills across different topic areas. Further research with larger samples will be needed to understand more fully individual differences in how home visitors respond to different scenarios.

Study limitations included a small sample size which may have reduced the ability to detect small effects. A post-hoc power analysis using *Gpower* (Faul et al. 2007) indicated that our sample of 51 participants at follow-up 2 yielded sufficient power to detect a medium to large effect at the recommended 0.80 level using simple multiple regression (Cohen 1988). However, we used a conservative analytic approach that adjusted for clustering within programs and thus reduced statistical power. In addition, although care was taken to balance content and difficulty level of the scenarios across time points, mock visits may have varied in perceived difficulty across assessments. Finally, self-reports of attitudes and confidence may have been prone to social desirability bias.

These findings provide empirical support for the acceptability and effectiveness of a trans-model approach to teaching motivational communication skills for use by home visitors when talking with families about sensitive issues. Future research could extend this work to examine how home visitors’ communication with families varies depending on the nature of the topic being discussed, and to test how communication strategies might be tailored when families present specific challenges, such as maternal depression or substance abuse. More research is needed to understand multi-level factors that influence training transfer and workforce performance, including supervision and coaching. To begin to address this, we are coding audio recordings of supervision sessions to examine the extent to which skills taught in the training were reinforced in supervision. More research is also needed to examine links between specific interpersonal skills and family outcomes.

We recommend that programs and researchers increase the use of observational measures of home visiting and consider mock visits as a tool by which to observe home visitor behaviors. Prior research has shown marked variability in communication skills in actual visits (Korfmacher et al. 2018). We know there is keen interest by programs in observational measures (Duggan and O’Neill 2016). Our experience suggests that these instruments have tremendous potential as tools for use in training, supervision, and assessment. Use of these methods and instruments in research and practice could be tested using the practice-based research network of the Home Visiting Applied Research Collaborative (2017). As the US continues to invest in the scale up of evidence-based home visiting, we must assure that workers are competent to communicate effectively about sensitive issues in the lives of the families served.
