# Correction to: Randomized Trial of a Training Program to Improve Home Visitor Communication around Sensitive Topics

**DOI:** 10.1007/s10995-018-2624-9

**Published:** 2018-08-22

**Authors:** Allison West, Laina Gagliardi, Amanda Gatewood, Susan Higman, Jane Daniels, Kay O’Neill, Anne Duggan

**Affiliations:** Department of Population, Family and Reproductive Health, Johns Hopkins Bloomberg School of Public Health, Baltimore, USA

## Correction to: Maternal and Child Health Journal 10.1007/s10995-018-2531-0

The article “Randomized Trial of a Training Program to Improve Home Visitor Communication around Sensitive Topics”, written by Allison West, Laina Gagliardi, Amanda Gatewood, Susan Higman, Jane Daniels, Kay O’Neill and Anne Duggan, was originally published electronically on the publisher’s internet portal (currently SpringerLink) on 31 May 2018 without open access. With the author(s)’ decision to opt for Open Choice the copyright of the article changed on 18 July 2018 to © The Author(s) 2018 and the article is forthwith distributed under the terms of the Creative Commons Attribution 4.0 International License (http://creativecommons.org/licenses/by/4.0/), which permits use, duplication, adaptation, distribution and reproduction in any medium or format, as long as you give appropriate credit to the original author(s) and the source, provide a link to the Creative Commons license and indicate if changes were made.

The original article has been corrected.

